# Engaging indigenous patient partners in patient-oriented research: lessons from a one-year initiative

**DOI:** 10.1186/s40900-020-00216-3

**Published:** 2020-07-22

**Authors:** Marie-Claude Tremblay, Maude Bradette-Laplante, Danielle Bérubé, Élaine Brière, Nicole Moisan, Daniel Niquay, Maman-Joyce Dogba, France Légaré, Alex McComber, Jonathan McGavock, Holly O. Witteman

**Affiliations:** 1 Department of Family Medicine and Emergency Medicine, Faculty of Medicine, Université Laval, Québec City, Canada; 2 Office of Education and Continuing Professional Development, Faculty of Medicine, Université Laval, Québec City, Canada; 3 VITAM, Centre de recherche en santé durable, Centre intégré universitaire de santé et de services sociaux de la Capitale-Nationale, Québec City, Canada; 4 Department of Family Medicine, Faculty of Medicine, McGill University, Montréal, Canada; 5 Department of Pediatrics and Child Health, Rady Faculty of Health Sciences, University of Manitoba, Winnipeg, Canada; 6 The Children’s Hospital Research Institute of Manitoba, Winnipeg, Canada

**Keywords:** Patient and public involvement, Patient engagement, Research involvement, Aboriginal, Indigenous, Minority groups

## Abstract

**Background:**

Patient-oriented research (POR) is a specific application of participatory research that promotes active patient engagement in health research. There is a growing concern that people involved in POR do not reflect the diversity of the population such research aims to serve, but are rather those more ‘easily’ engaged with institutions, organizations and society. Indigenous peoples are among such groups generally underrepresented in POR. The “Indigenous patient partners platform project” was a small-scale initiative aimed to address the issue of the underrepresentation of Indigenous people in patient-oriented research by recruiting, orienting and supporting Indigenous patient partners in Québec (Canada). This article reports on the findings of an evaluation conducted at the end of the project to garner lessons and identify strategies for engaging Indigenous patient partners in patient-oriented research.

**Methods:**

The evaluation of this initiative used a case study design hinging on documentary analysis and committee member interviews. Project documents (*n* = 29) included agendas and meeting minutes, support documents from the orientation workshop and workshop evaluations, and tools the committee developed as part of the project. Interview participants (*n* = 6) were patients and organizational partners. Thematic analysis was performed by two members of the research team. Patient partners actively contributed to validating the interpretation of result and knowledge translation.

**Results:**

Results point to four key components of Indigenous patient partner engagement in POR: initiation of partnership, interest development, capacity building and involvement in research. Specific lessons emphasize the importance of community connections in recruiting, sustaining and motivating patient partners, the need to be flexible in the engagement process, and the importance of consistently valuing patient partner contributions and involvement.

**Conclusions:**

There is a need to engage Indigenous patient partners in POR to ensure that healthcare practices, policies and research take their particular needs, stories and culture into account. While results of this evaluation are generally consistent with the existing literature on patient engagement, they offer additional insight into how to effectively engage Indigenous patient partners in research, which might also be relevant to the involvement of other marginalized populations who have been historically and systemically disempowered.

## Plain English summary

‘Patient-oriented research’ is a type of health research that actively involves patients, their caregivers, and their families as partners (i.e. patient partners) in the research process. Some people or groups are less likely to take part in research due to a variety of socioeconomic, cultural, or societal reasons. Thus, their views, needs and perspectives are often not considered when making decisions regarding research topics or projects. Indigenous peoples in Canada (First Nations, Inuit and Métis) are less often involved as patient partners in research. The “Indigenous patient partners platform project” is an initiative that aimed to recruit, orient, engage and support new Indigenous patient partners in health research in Québec (Canada). At the end of the one-year period we evaluated the initiative to identify the best strategies for engaging Indigenous patient partners in patient-oriented research. The results of the evaluation identified strategies related to how we initiate contact with patient partners, how we develop their interest for research, how we support their partnership in research and how we can genuinely involve them in research. Guidance as to how best engage Indigenous patient partners in research includes the importance of considering patient partners’ connections with their community; the need to be flexible in the engagement process to account for Indigenous patients’ diversity; and the importance to understand the historical and social context in which engagement occurs for Indigenous patient partners.

## Background

### Indigenous patient involvement in research

Research and healthcare approaches are increasingly drawing on participatory approaches that involve patients, health professionals and researchers [1–4]. Patient-oriented research (POR) is an approach used to promote active patient involvement in health research [5]. ‘Patient partner’ is an overarching term given to individuals who have a personal experience with a health issue, whether as patients themselves or as informal caregiver, and who play an active and meaningful role in governance, priority setting, conducting research and knowledge translation [5]. POR has the potential to increase research quality, validity and relevance, while improving health outcomes for the general population [2, 6–12]. Participatory research approaches such as POR are preferred when working with groups of people who have historically had less decision-making power and access to health research [13–17], as they facilitate power sharing between researchers and those researched (i.e. patients), and acknowledge the legitimacy of experiential knowledge to promote action research. In recent years, POR has been promoted in many industrialized countries through national policies and major public investment in research institutes and networks (e.g. INVOLVE in the UK; Patient-Centered Outcomes Research Institute in the United States; Strategy for patient oriented research in Canada), as well as thematic funding opportunities [18].

There is a growing concern that people involved in POR do not reflect the diversity of the populations that research aims to serve [16, 17, 19, 20]. Instead, patient partners (i.e. patients actively engaged in research governance, priority setting, conducting research and knowledge translation) represent those who are more ‘easily’ involved with institutions, organizations and society; reinforcing existing societal power imbalances. Members of more marginalized populations are seldom represented among patient partners involved in POR [16, 17, 19]. As a result, their particular perspectives and needs are less considered in the decision-making processes around research results, which in turn reinforces their marginalization [20]. There is a need to broaden the range of people and experiences involved in POR. More creative ways to engage populations characterized as ‘hard to reach’ (but might in fact be ‘overlooked’ or ‘ignored by society’) are needed to meet this need [16, 17]).

Indigenous peoples are among those groups of people less involved with mainstream institutions, and who remain generally underrepresented in POR [16]. In Canada, Indigenous people experience considerable health inequities compared to the general population [21–23]. For instance, adults living on First Nations reserves have much higher rates of type 2 diabetes and associated complications compared to the national adult population [21]. Some of these inequities, including diabetes, are associated with colonialism, social exclusion and discriminatory practices experienced by Indigenous peoples [23–26]. It is through genuine and equitable participation that Indigenous communities and peoples can take action for change, self-determination and empowerment [27]. There is a long history of equitable partnership with Indigenous peoples in community-based intervention/research in Canada [28–30]. Building on this legacy, engaging Indigenous people in POR is crucial to amplify their voices in health research and policies. However, we know little about the most effective strategies to increase POR with Indigenous patient partners. This paper reports on findings of an evaluation of the “Indigenous patient partners platform project”, a mobilization initiative aimed to address the issue of underrepresentation of Indigenous people in POR.

### Description of the initiative: the indigenous patient partners platform project

The “Indigenous patient partners platform project” aimed at recruiting, orienting and supporting Indigenous patient partners to play an active role in health research in Québec (Canada), and more specifically within a national diabetes research network. This one-year engagement initiative used a participatory approach involving researchers, Indigenous people living with diabetes, as well as Indigenous and healthcare organizations in the province of Québec.

As a first step in the project, we created a committee in May 2017 consisting of organizational partners and research team members, to define the goal and procedures of the initiative (see Table 1 for project partner sociodemographic). Organizational partner representatives (*n* = 6) included health professionals and decision makers from Indigenous organizations and health organizations serving Wendat, Atikamekw and Maliseet communities, as well as one member of a Native Friendship Center. This group of partners helped recruit 8 Indigenous patient partners (4 men and 4 women) living with diabetes and identify strategies to empower patients in their roles as patient partners. Three patient partners were Maliseet from Viger living off community; three were from the Atikamekw community of Manawan; one was Abenaki from Odanak; and one was Innu from Pessamit but living in Trois-Rivières (Table 1). From June 2017 to June 2018, project members including research team members, organizational partner representatives and patient partners, met eight times mostly via teleconference. Meetings were held in French, which is Québec province’s official language and a language common to all project partners. Patient partners were compensated for their involvement in project meetings and the evaluation. As this project also comprised a qualitative evaluation study involving project partners as participants, this project was reviewed and approved by the Laval University Institutional Review Board (no. 2017–116).

**Table 1:** Project partners

Organizational partners	Location and clientele	n
Centre de santé Marie-Paule-Sioui-Vincent	Wendake, providing health services to patients from Wendake (Wendat)	2
Centre d’amitié autochtone de Lanaudière	Joliette, providing services to a diverse Indigenous clientele living in the area of Lanaudière	1
Centre de santé Masko-Siwin	Manawan, providing health services to the community of Manawan (Atikamekw)	1
Première Nation Malécite de Viger	Viger, providing services to the Maliseet population	1
Groupe de médecine familiale universitaire St-Charles-Borromée	Joliette, providing health services to the community of Manawan (Atikamekw)	1
**Patient partners**	**Location**	**n**
Atikamekw	Manawan and Joliette	3
Maliseet	Bic, Rivière-du-Loup and Québec	3
Abenakis	Odanak	1
Innu	Trois-Rivières	1

We used a participatory approach in co-developing the specific objectives, mission and strategies of the Indigenous patient partners platform project during the first committee meeting. The committee identified the following long-term objective for the initiative: to increase the number of respectful and culturally-relevant POR projects. With that long-term goal in mind, we agreed to form a core team of Indigenous patient partners living with diabetes and available to actively participate in future research projects as the medium-term goal. Short-term objectives were to define the vision, action plan and deliverables (i.e. tools for patients) of the project, all aimed at forming and sustaining the Indigenous patient partner group. The mission of the project was: “Giving a voice to Indigenous patients in research concerning them”.

After the second meeting, committee members decided to use an advertisement to recruit patient partners. While the flyer was useful for synthesizing information about the initiative, patient partners were generally recruited by word of mouth, and through contact persons in partnering organizations. After the third meeting, newly recruited patient partners (the core team of 8 patient partners described above) joined the committee. During the fourth and fifth meetings, the research team invited several presenters to inform the committee’s understanding of POR and the roles of patient partners. An experienced patient partner living with arthritis presented on his long-term engagement in research. We also invited various researchers to present and offer potential involvement opportunities for patient partners, such as a patient-oriented research network in diabetes, and specific research projects related to diabetes and/or Indigenous health.

The project culminated in a one-day orientation workshop, which aimed to reinforce project members’ capacities regarding POR. This workshop was developed in collaboration with the Québec provincial Strategy for Patient-Oriented Research support unit. The workshop aimed at giving both patient and organizational partners basic notions about health research, POR and patient partner roles (research team members were trained in POR research methods prior to the beginning of the project). An opening ceremony with an elder from a local community started the day and participants were introduced to relevant subjects regarding research project development, research ethics and funding. Different levels of patient involvement in research were also presented, as well as various patient partner profiles and several roles and responsibilities they can have in research.

Following the workshop, two other meetings took place aimed at identifying patient partners’ needs related to their new role, and to developing new engagement tools as required. Patient partners identified two useful tools, namely a project presentation form that had to be filled by researchers and a participation evaluation form, which allowed patient partners to evaluate their experience participating in a research project (Appendix 1 and 2).

## Methods

### Design

The evaluation of the Indigenous patient platform project aimed to garner lessons from the initiative and identify optimal strategies for engaging Indigenous patient partners in POR. The evaluation used a case study design [31] that hinged on qualitative methods, involving documentation analysis and committee member interviews. The case was bound by the initiative itself, that is from the first meeting in June 2017 to the last in June 2018. Patients partners recruited and trained through the initiative were involved in the last steps of the evaluation: the interpretation of results and knowledge translation.

### Data sources

At the end of August 2018, project partners were sent an email inviting them to take part in qualitative interviews. Of the 12 partners contacted, 6 accepted. The main reason given for non-participation were a lack of time or insufficient knowledge about the project (this was a reason evoked by organizational partners who participated more at the beginning of the project). Two of the patient partners did not provide answer. Patient partners (*n* = 4; one man and three women, from Maliseet and Atikamekw nations) and organizational partners (*n* = 2; two women, from two organizations related to the Atikamekw nation) (see Table 2) were interviewed individually in September 2018, about 3 months after the last team meeting. The interviews were meant to identify strengths and weaknesses of the initiative, as well as the barriers and enablers to Indigenous patient engagement in research. The team developed questionnaires according to these objectives, and included 14 open-ended questions asking participants about their experience in the project, how their needs were considered, and how the initiative could be improved. The interviews were conducted in French by teleconference or in person, by a research professional (MBL) who joined the project at the end of the one-year period. Each interview lasted an average of 30 min. All participants provided written consent.

**Table 2:** Interview participants

Participants characteristics	
Type of partners	
Patient partners	4
Organizational partners	2
Gender	
Men	1
Women	5
Nations	
Maliseet	3
Atikamekw	3

Documentary analysis focused on internal project documents and included agendas and meeting minutes (*n* = 16), support documents from the orientation workshop and workshop evaluations (*n* = 10), and the tools developed as part of the project (*n* = 3).

### Data analysis

A professional transcriptionist transcribed interviews verbatim and transcripts were verified by a research team member (MBL) for accuracy. All transcripts and project documents were integrated into a database in NVivo 12. Two team members (MCT and MBL) performed inductive thematic analysis [32]. Two coders (MBL and MCT) generated the initial codes and themes related to strengths and weaknesses of the initiative, and facilitators and barriers of engagement. The two coders compared themes and resolved any coding differences through consensus. All patient partners recruited through the project were invited to take part in the last steps of the evaluation. Four of the patient partners, the same who had taken part in the qualitative interviews, accepted. This process served as a form of member check and contributed to validate the interpretation of results. Working with the research team, they took part in a collective discussion focused on interpretating results, and helped create a presentation of results for conferences and this paper. One of the patient partners also presented the results of this project at a conference.

## Results

The evaluation results offer lessons for how to build meaningful partnerships with Indigenous patient partners in research. Results point to a number of strategies pertaining to the four components of patient engagement: initiation of partnership; interest development; capacity building; and involvement in research.

**Fig. 1:**
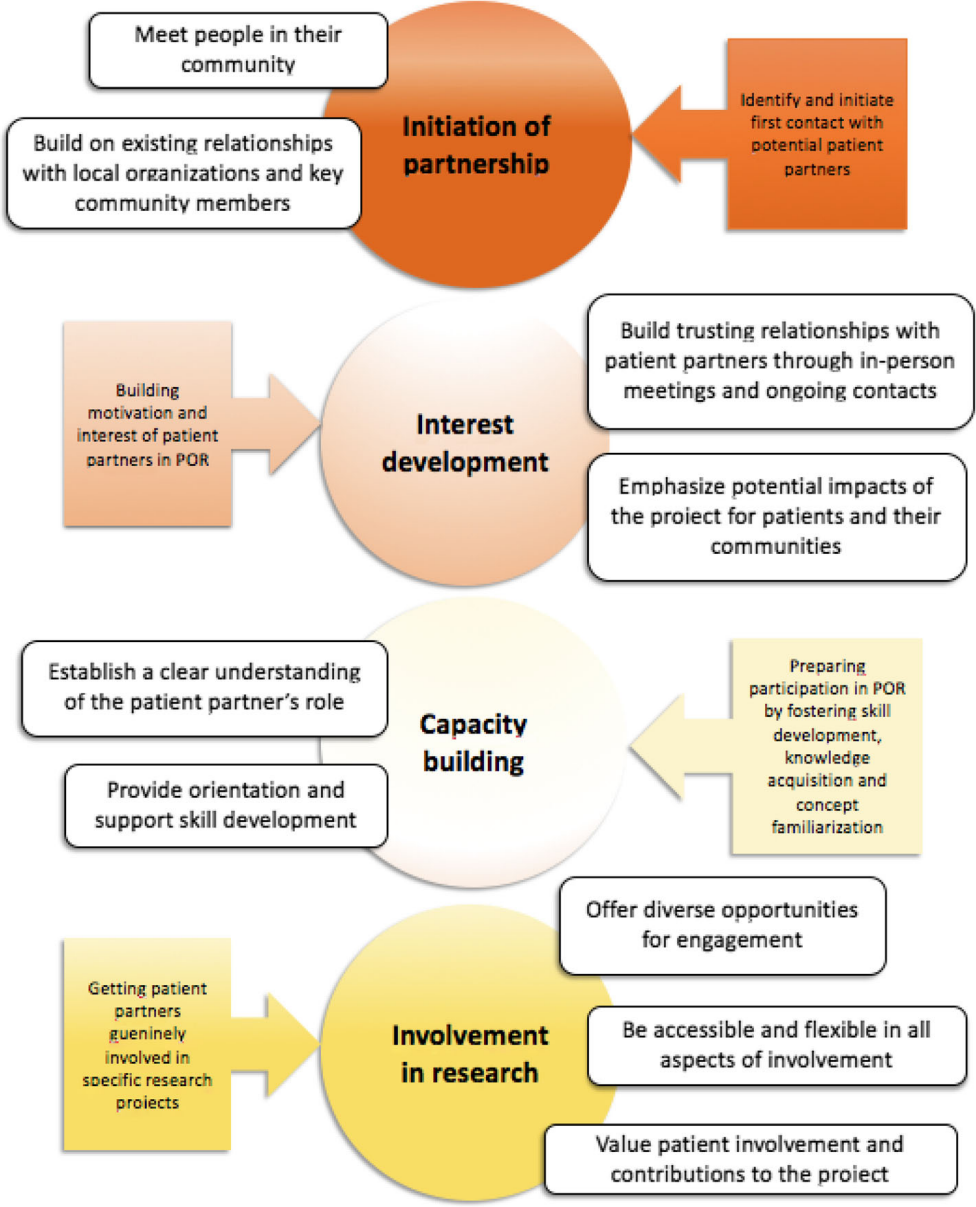
Four components of Indigenous patient engagement in research

The specific themes that emerged within each of the four components are presented in Fig. 1.

### Initiation of partnership

#### Meet people in their communities

Recruiting Indigenous patient partners is a crucial first step in involving patients in POR. It involves finding effective ways to identify and approach potential patient partners to interest them in research. In this project, we learned the importance of initiating first contact with potential Indigenous patient partners in their communities. Due to budget constraints, this project relied mostly on remote contact (teleconference) with participants, which was perceived by patient partners and organizational partners as a main limitation to recruitment. Hence, project members unanimously agreed that meeting people in person in their own community would facilitate initial contact and relationship building with potential patient partners. One participant explained: “Maybe it’s going in the communities, meeting them ( …) then explaining well what the mission is, what you do in your project” (Interview, PP).

#### Build on existing relationships with local organizations and key community members

Apart from going directly to communities, leveraging existing relationships with local organizations, community leaders or trained patient partners can also facilitate contact with potential patient partners. In fact, one of the initiative’s main strategies was to link with organizational partners to identify and approach potential patient partners according to the initiative’s needs. In addition, patient partners can act as ambassadors by promoting the importance of POR and recruiting other patient partners in their community: “I think it is word of mouth, but that comes from them, so that a patient who is already a patient partner, I think it’s (…) the best person to go recruit, because they will have the experience, they will see how it works (…) (interview, PP). For example, during this project, one of the participants acted as an ambassador by recruiting her two cousins.

### Interest development

#### Build trusting relationships with patient partners through in-person meetings and ongoing contact

Another finding is that building and sustaining the interest of patient partners in POR relies on using a number of mechanisms to ensure repeated personal contact and continued relationships. The in-person training workshops, where patient partners got to connect face-to-face, facilitated bonding between the patient partners, the organizational partners and the research team: “The meeting [workshop] where we were really all together; where we could look at each other in the eyes - I would say, and connecting with one another. Then the fact that we had lunch together, that was fun, and it changed the conversation” (interview, PP). Sustaining interest also involved frequently checking in, and maintaining ongoing contact with patients by phone or email. In addition, one of the patient partners was offered the opportunity to attend a conference and present the project together with the main researcher, which tightened the bond between them. The patient was surprised to realize that she could truly be an asset to the researcher, and the experience strengthened their mutual trust: “And also what I liked, it’s [the principal researcher]‘s trust. Because when we went to Toronto, she had someone who was supposed to help her, but that person was unable to make it. So then she said, ‘Can you help me?’ She trusted me. I enjoyed that″ (interview, PP).

#### Emphasize potential impact of the project for the patients and their communities

While POR does not necessarily focus on the communities in which people live, it is important to explain the potential impact of POR research projects for patient partners’ communities, in order to ignite interest and motivation in the research. For example, some Indigenous patient partners have a strong bond with their community, and were particularly interested in how the initiative could benefit their community. As an organizational partner highlighted: “I think there are people who are always a little bit worried to embark on things like this. ( …) Always worried what it is going to do, what it is going to bring them and their community in the end.” (interview, OP). Hence, their engagement in research projects can be improved by explaining how the results of the project could benefit their community at the end.

### Capacity building

#### Establish a clear understanding of the patient partner’s role

Capacity building involves fostering skill development, knowledge acquisition and familiarization with conceptual tools needed for patient partners to enact their role. In this initiative, the ‘patient partner’ concept was central to our endeavour, but was a difficult concept to grasp for some, constantly requiring explanation and examples. As a result, we had several presenters exemplify various roles of patient partners to familiarize them with the concept. For most patient partners, the idea was initially very unclear, as one participant stated, *“*It’s certain that at the beginning I was lost, really lost. And then I was asking myself ‘what am I doing here’?” (interview, PP). Despite this initial challenge, participants came to better understand the patient partner role over time. Most of them noted that the repeated explanations, presentations, and particularly their involvement in projects helped them progressively understand the definition and roles of patient partners. This ultimately helped increase their capacity to take on the role.

#### Provide orientation and support skill development

Capacity building was a continual process, but for most patient and organizational partners, the one-day workshop they attended was a pivotal moment (from the interviews). One patient partner stated in the workshop evaluation comments, “Now, I really understand the concept of patient partner”. In this project, we found that skill development and knowledge acquisition proved to be central components as many patient partners initially lacked confidence in their capacities. Through positive feedback and positive reinforcement, the research team helped participants build confidence and express themselves openly, which heightened their level of involvement. One participant explained: “I was even surprised sometimes they said ‘Yes that’s a great question. We will look into that’; We could see that the team was happy that we participated ( …)” (interview, PP).

### Involvement in research

#### Offer diverse opportunities for engagement

Getting patient partners genuinely involved in specific health research projects is the ultimate goal of patient engagement. Throughout the initiative, patient partners were offered a number of opportunities for engagement in external research projects. This allowed them to appreciate the spectrum of projects they could engage in and helped them better understand what POR was. This was also one of the main strategies used to strengthen the impact of the initiative. By integrating patient partners in actual research teams, we aimed to foster their involvement as a patient partner in research. A patient who was involved in an external research project mentioned that her involvement in a project allowed her to apply the information she had obtained from the orientation workshop (meeting minutes).

#### Be accessible and flexible in all aspects of involvement

Being accessible means providing full opportunity for all team members to be involved and participate. Throughout the initiative, patient partners experienced different life challenges (e.g. death in the family, being a caregiver for a family member, or receiving extensive treatments for their disease) or had obligations that prevented them from attending some of the meetings. The committee purposely scheduled meetings according to patient partners’ availability. In the interviews, some participants mentioned that they appreciated that the research team was flexible with time and felt like their own schedule was considered. This level of accommodation increased their willingness to be involved.

Access to communication technology is also important to consider, especially for partners located in remote communities and/or with limited Internet or telephone access. For instance, one patient partner had no access to a personal phone or the internet. In this case, an organizational partner acted as a resource person by providing office space and telephone access so that the patient partner could participate in meetings.

Being accessible also involves adapting to different levels of scientific or academic literacy. Researchers and health professionals often use technical or scientific jargon that can create discomfort and misunderstanding and make patient partners feel that they are not central members of the research team. For instance, in this initiative, the invitation letter required by the Institutional Review Board was, according to a patient partner, “completely incomprehensible ( …) people in his family did not understand anything when reading the form” (from the meeting minutes, PP). With the Institutional Board’s approval, the initial invitation letter was revised to ensure it was written in plain language, and was understandable to Indigenous patients who might not have French as their mother tongue.

#### Value patient involvement and contributions to the project

The time and energy patient partners give to a research project is an invaluable asset to research teams. Letting patient partners know that they are appreciated and that their contributions impacted the project seemed to be crucial to foster their involvement in other projects. For instance, a patient mentioned that feeling valued was a great motivator for engaging in projects: “This may be why I now look forward to getting involved in a new project - to feel valued” (interview, PP). Valuing patient partners’ involvement also implies offering them financial compensation for their time and work. One patient partner revealed that receiving compensation for her involvement was an interesting benefit (interview, PP). The importance of valuing patient partners’ time and contributions is especially important considering how some patients experienced low self-confidence within this initiative. In the interviews, one of the patient partners confided that she was afraid that the time invested in her was worthless: “And then later on I understood, I thought ‘so they are doing recruitment’. So, then I started being afraid that you invested in me, because now I get compensated, you take time to train me, but what if I don’t do the job?” (interview, PP). The research team showed participants that their involvement in this initiative was of great importance by expressing gratitude and providing positive feedback.

### Patient feedback

As mentioned, all patient partners involved in the project were invited to a follow-up meeting where results of the project were presented and discussed. Four patient partners who had taken part in the interviews accepted the invitation and attended the meeting. All patients felt that the results represented generally well their experiences in the project, as well as what they said in the interviews. They find the four components of patient engagement (i.e. initiation of partnership, interest development, capacity building and involvement in research) relevant, as well as the strategies associated with each. Some patients questioned the wording utilized. For instance, one patient partner found the word ‘engagement’ unclear, and suggested to define it. Patients also offered advice related to knowledge translation of the results to organizational and community partners involved in the project.

## Discussion

This project is one of the few to address the issue of underrepresentation of Indigenous people in POR. It provides guidance and discusses specific strategies to engage Indigenous patient partners in patient-oriented research. Results of this evaluation are generally consistent with the existing literature on patient engagement in research. For instance, the importance of building trusting relationships from the beginning of the process and through in-person meetings has been noted by many [6, 7, 17, 33]; particularly in the case of groups who are ‘seldom-heard in research’. Flexibility in the engagement process is also emphasized to promote inclusion and diversity in POR [16, 17]. Additionally, fostering a clear definition of the patient partner role, providing financial support and training, valuing patient involvement, and recognizing participant contributions are deemed essential elements to meaningful involvement in research [33, 34] and are usually considered best practices for patient engagement [6, 7, 35, 36].

Our results also point to elements that are specific to the engagement of Indigenous patient partners, such as the importance of community connections and ties in recruiting, sustaining and motivating patient partners. As many Indigenous people have deep ties to their communities which have historically been excluded, meeting potential patient partners on their own grounds is particularly important. Additionally, building on existing connections with trusted local organizations or community champions seems a relevant strategy. Similarly, it is important for patient partners, to be able to link their involvement in a given research project to its impact for their community. For instance, a recent study found that the main impetus for patient partners engagement in research is benefiting others [37]. In the case of Indigenous patient partners in our initiative, this altruistic motivation was specifically oriented towards their cultural community. This finding highlights the importance of integrating into POR Indigenous models of health research in which individuals are not separable from their communities. When patient partners are Indigenous, patient-oriented research may need to better bridge the gap between ethical principles for standard health research and ethical principles for community-based research with Indigenous communities (e.g., Ownership, Control, Access and Possession principles for research with First Nations communities.)

Another important lesson is being flexible enough to accommodate different levels of knowledge and access to resources (financial, technological, literacy), which can be challenging when working with patients from diverse backgrounds and communities. For instance, in this project some patients were living in communities in remote areas and others were living in urbanized settings, all with different financial means and access to communication technology. These results are especially important to guide strategies for engaging Indigenous patient partners in future POR projects.

### Reflecting on challenges encountered

While the results emphasize enablers and strategies to Indigenous patient partner engagement, we also experienced challenges in this endeavor. Based on our experience in this project, here are some specific issues related to engaging Indigenous patient partners that were grounded in cultural, structural and institutional factors. The first challenge was to not consider Indigenous populations as a homogenous ethnic group. Not only do they have different cultural backgrounds, they also have different levels of engagement with their own culture, traditions and beliefs. In this project, patient partners came from four different communities and nations, all with different level of knowledge of, and attitudes toward, their culture. This reinforced the importance of learning and knowing the cultural backgrounds, traditional beliefs and social customs of the people with who we were interacting [17]. Working with a diverse group of patients triggered the need to honor diverse beliefs, traditions and needs of Indigenous patient partners, while creating a common safe space to encompass this diversity. Also, engaging Indigenous patient partners across diverse communities meant employing different strategies and a broader network of individuals and organizations.

A second challenge that we experienced was to fully understand and appreciate the influence of historical and social context in which engagement occurs for Indigenous patient partners. Indigenous peoples across Canada share a similar history of oppression and marginalization related to colonization, which may negatively affect their attitudes or relationships with institutions and research. In this initiative, some of the participants lacked self-confidence in their capacity to participate; some even felt like imposters in their patient partner role and questioned their ability to do the work. This lack of self-confidence working within Western-dominated organizations and world views is a form of historically-rooted disempowerment resulting from colonialism. Accordingly, some patients in this initiative did not trust institutions and were reluctant to provide personal information to the university. Similarly, many participants had difficulty understanding the concept and the role of a patient partner. Being an active actor in a Western-dominated process and organization challenges the Western standard view of the patient as passive and vulnerable [38]. All this points to the need to be conscious of the legacy of power dynamics that may affect Indigenous patient partners during the engagement process. To this end, we found it useful to equalize the power dynamic in the committee as much as possible by calling people by their first names instead of titles (a strategy that has also been identified by others [33]), to favor open and welcoming working practices, and to frequently value and provide positive feedback on patients’ involvement.

The third challenge is that, despite the increasing popularity of POR, our institutions are ill-prepared to manage patient partnership in research, and the existing barriers for patient partners can be greater for Indigenous participants. For instance, in this project, the financial compensation payments and expense reimbursements took many weeks to process. This kind of delays is not always justifiable from a patient partner perspective and is especially hard to endure for financially disadvantaged patients. Another barrier at our institution is that, there is no relevant accounting code to categorize patient partner contributions. They can be treated as “experimental subject” (in which case they can receive non-recurring financial compensation, which should not exceed 100$ and is not taxable); otherwise, they are treated as institutional employees receiving a taxable salary. These two scenarios pose serious challenges for some Indigenous patient partners who are receiving social assistance and therefore cannot receive more than a certain amount in donations or salary per month. Also, in this initiative, some of the patient partners did not have an email address nor a bank account number, which are usually required by the institution for payment deposits. In this case, we had to struggle with the institution’s financial services to find an alternative solution. All these considerations lead to the conclusion that there is a crucial need to rethink how patient partner are compensated. This reflection needs to take place at the institutional and policy levels, in order to define more relevant ways to characterize and value citizen engagement in research. To this effect, financial compensation policies, developed in partnership with patient partners, can be useful to clearly define how, how much, for who, on what occasions financial compensation can be offered to patient partners.

Apart from administrative barriers, there are also concerns regarding the ethical assessment of these types of projects. While the frontier is thin, research with Indigenous patient partners is different from participatory community-based research with Indigenous communities (as mentioned earlier), and guidelines in both cases are different. For instance, principles of ownership of data and results, control, access and possession don’t necessary apply in the case of a POR research project involving Indigenous patient partners from different communities. This can be confusing for ethics committees, which are still new to the concept and also sometimes to research with Indigenous partners. In this initiative, the ethics committee was initially opposed to providing tobacco as a gift for elders at the workshop in keeping with cultural protocols, which highlights the need to sensitize institutional boards about cultural aspects of research with Indigenous patients and communities.

### Limitations

The results presented here relate to a small-scale strategic engagement initiative in Québec (Canada) and should be interpreted in this context. Conclusions build on project documentation and a limited number of perspectives. More specifically, interview participants were those who were most highly involved in this one-year project. Their views may not represent that of other project partners, as they were more committed to the project. There may be a certain level of social desirability bias in the interviews, even though the interviewer strove to maintain neutrality throughout the interview process. Patient partners in this project experienced several life challenges (a death in the family; being a caregiver for a family member; change of residence). As a consequence, four patient partners were not able to complete this project. Despite our efforts to sustain the relationships with patient partners, this has proved difficult, and is perhaps part of the challenge of establishing partnership with members of populations living in tenuous conditions.

## Conclusion

Engaging Indigenous patient partners in research is needed to ensure that their needs, experiences, knowledge and culture are better integrated in all aspects of health care and research. This project aimed at recruiting, orienting and supporting Indigenous patient partners in Québec (Canada), to play an active role in health research. An evaluation of this small-scale initiative was carried out to garner lessons and identify the most effective strategies to increase Indigenous involvement in POR. Results provided lessons pertaining to the four components of patient engagement: recruitment, engagement building, capacity building and involvement. While results of this evaluation are generally consistent with existing literature around patient engagement, they additionally offer important insights into how to effectively engage Indigenous patient partners in research. Specific lessons emphasize the importance of community ties to recruit, sustain mobilization and motivate patient partners; the need to be flexible in all aspects of the engagement process; and the importance of constantly valuing patient partner contributions and involvement. Challenges experienced through this initiative highlight the needs: to create a common safe space to encompass Indigenous patients’ diversity; to understand the influence of historical contexts and colonialism in which Indigenous patient partners are engaging; and to heighten the capacity of our institutions to work with these kinds of partnership.

## Supplementary information

**Additional file 1.** Presentation Sheet - Description of research projects.

**Additional file 2.** Evaluation Sheet - Evaluation of participation.

## Data Availability

Due to the small number of participants involved in the evaluation of this project and to sensitive information provided by participants regarding their health, the datasets generated and analyzed during the current study are not publicly available to protect the confidentiality of participants.
