# Supplementary material for: Engaging indigenous patient partners in patient-oriented research: lessons from a one-year initiative
Source: Res Involv Engagem. 2020 Jul 22;6:44. doi: 10.1186/s40900-020-00216-3 (PMC7376932; doi:10.1186/s40900-020-00216-3)
Supplement: Supplementary file 1 — Additional file 1. Presentation Sheet - Description of research projects. [file 40900_2020_216_MOESM1_ESM.docx]

| Research project sheet | |
| --- | --- |
| Project title |  |
| Name and contact information of the person in charge of the project |  |
| Project description (goal, steps) |  |
| Involvement of patient partners in the project (type of involvement, number of patient partners, time required, etc.) |  |
| Benefits of participating as a patient partner |  |

**Instructions to researchers:**

- Use accessible and simple language.
- Clearly define scientific or complex terms.
- Avoid the use of acronyms or abbreviations.
- Use short sentences (one idea per sentence).
- Clearly describe the nature of involvement of the patient partner.
