# Supplementary material for: Engaging indigenous patient partners in patient-oriented research: lessons from a one-year initiative
Source: Res Involv Engagem. 2020 Jul 22;6:44. doi: 10.1186/s40900-020-00216-3 (PMC7376932; doi:10.1186/s40900-020-00216-3)
Supplement: Supplementary file 2 — Additional file 2. Evaluation Sheet - Evaluation of participation. [file 40900_2020_216_MOESM2_ESM.docx]

**Evaluation Sheet - Participation as a patient partner**

| 1 | Project name |  |
| --- | --- | --- |
| 2 | Name of lead researcher |  |
| 3 | Have you ever been involved in a research project before? | Yes / No |
| 4 | What encouraged you to participate in this research project? |  |
| 5 | Were the project and your role explained to you before you began to participate? | Yes / No |
| 6 | Was your schedule taken into consideration when planning the meetings? | Yes / No |
| 7 | What kind of financial compensation did you receive for your participation? | Gift card  Fee for service or one-time financial compensation  Regular salary or regular payments throughout the duration of the project |
| 8 | What could the research team have done to improve your experience? |  |
| 9 | What did you learn from working with the research team? |  |
| 10 | Did you feel comfortable speaking at research team meetings? | Yes / No  Please explain: |
| 11 | Do you feel that you contributed to the study? | Yes / No  Please explain: |
| 12 | Would you like to participate in another research project? If so, why? If not, why? | Yes / No  Please explain: |
